# EXPECTATIONS AND VALUE OF EDUCATION OF STUDENTS OF THE MASTER IN GERONTOLOGY OF THE UNIVERSITY OF GUADALAJARA

**DOI:** 10.1093/geroni/igz038.2656

**Published:** 2019-11-08

**Authors:** Martha Elena Vazquez Arias, Elva Dolores Arias Merino

**Affiliations:** Centro Universitario de Ciencias de la Salud. Universidad de Guadalajara, Guadalajara, Jalisco, Mexico

## Abstract

For Institutions of Higher Education, it’s important to know better the expectations and the value of education of their students to plan and offer successful postgraduate educational services The purpose of this project is to identify and analyze the expectations and the value of education of students of the Master in Gerontology at the University of Guadalajara, The data collection was carried out through a questionnaire divided into two parts. The first one contemplated socio-demographic characteristics of the participants such as: name, age, marital status and coexistence with older adults. In the second part, the Value of Education Scale (Battle & Wigfield, 2003) was applied, which measures different aspects of the expectations and the value that graduate students give to education. 25 students participated. 64% (16) were women and 36% (9) were men, with an age range of 24 to 53 years. Single people (80%) and 56% coexist with older adults every day. Highest scores: Intrinsic Values “I like to learn from people who are experts in their field” 4.96. Value of Achievement “graduate studies have a great value for me” 4.64. Value of Utility “I want to complete my postgraduate studies to acquire knowledge and experience” 4.80. Cost Perceived “I think in the end it will be worth the postgraduate despite all the work and effort.” 4.96. We consider a guarantor of success for our students, their high expectations and the value they give to their education that make them stand out and gain knowledge every day.

